# Critical Roles of IL-33/ST2 Pathway in Neurological Disorders

**DOI:** 10.1155/2018/5346413

**Published:** 2018-02-04

**Authors:** Mohammad Faruq Abd Rachman Isnadi, Voon Kin Chin, Roslaini Abd Majid, Tze Yan Lee, Maizaton Atmadini Abdullah, Ramatu Bello Omenesa, Zaid Osamah Ibraheem, Rusliza Basir

**Affiliations:** ^1^Department of Human Anatomy, Faculty of Medicine & Health Sciences, Universiti Putra Malaysia, Serdang, Malaysia; ^2^Department of Medical Microbiology & Parasitology, Faculty of Medicine & Health Sciences, Universiti Putra Malaysia, Serdang, Malaysia; ^3^Department of Pathology, Faculty of Medicine & Health Sciences, Universiti Putra Malaysia, Serdang, Malaysia

## Abstract

Interleukin-33 (IL-33) is an IL-1 family member, which exhibits both pro- and anti-inflammatory properties solely based on the type of the disease itself. Generally, IL-33 is expressed by both endothelial and epithelial cells and mediates its function based on the interaction with various receptors, mainly with ST2 variants. IL-33 is a potent inducer for the Th2 immune response which includes defence mechanism in brain diseases. Thus, in this paper, we review the biological features of IL-33 and the critical roles of IL-33/ST2 pathway in selected neurological disorders including Alzheimer's disease, multiple sclerosis, and malaria infection to discuss the involvement of IL-33/ST2 pathway during these brain diseases and its potential as future immunotherapeutic agents or for intervention purposes.

## 1. Introduction

IL-33, also designated as IL-1F11, is a newly identified cytokine which belongs to and is classified as the 11th member of the IL-1 family [1]. It was first identified as DVS27, a highly upregulated gene found in canine vasospastic vertebral arteries [2], and later as the nuclear factor from high endothelial venules, NF-HEV [3], prior computational analysis by Schmitz and coworkers, which leads to the reidentification as IL-33 due to the sheer similarity with IL-1 family members [1]. IL-33 initiates its signalling pathway by binding to the ST2 receptor, or also known as IL-1R4, with variants which are comprised of ST2L, sST2, ST2V, and ST2LV [1, 4–7]. Apart from ST2, the interaction of IL-33 with a single immunoglobulin IL-1R-related molecule (SIGIRR) has been documented as well [8, 9]. With exception from other IL-1 family members, IL-33 is primarily involved in the Th2 immune response induction [1] which confers protective immune response towards parasitic infection, specifically helminths [10–12], and deleterious immune response towards hypersensitivity or allergic reactions, especially asthma [5, 13, 14].

IL-33 and its role has been widely investigated in numerous diseases particularly but not limited to the various degrees of hypersensitivity-related and infectious diseases, whereby several of the diseases are confined within the brain [15–17]. IL-33 is identified as a “dual cytokine” by exerting its protective and deleterious effect, either as a pro- or anti-inflammatory role, and based on acting as a regular cytokine or nuclear transcription factor, which depends upon the model and the disease [18] and type of cells [1, 19–22], hence appearing to be a “double-edged sword” [23]. There are several insights that point out the potential of IL-33/ST2 pathway for novel biomarker discovery and therapeutic target in diverse brain diseases [16, 24, 25]. Therefore, in this review, the critical roles of the IL-33/ST2 pathway in neurological disorders are discussed, particularly in Alzheimer's disease, multiple sclerosis, and experimental cerebral malaria.

## 2. Biological Features and Structure of IL-33

IL-33 is a cytokine which exerts its dual functions on two mechanisms: either as an extracellular “traditional cytokine” or is considered as “intracellular nuclear factor with transcriptional regulatory properties” [26]. IL-33 mRNA or protein can be found in various types of organs by a multitude of cells including endothelial and certain epithelial cells [1, 20], mast cells [21], activated macrophages, and dendritic cells [1]. As a cytokine, IL-33 binds to ST2L/IL1-RAcP complex by inducing Th2 cytokines and chemokines through polarised Th2 cells, such as alternatively activated macrophages (AAM or M2) [27–32]. Despite the functional role of IL-33 as a nuclear transcription factor is yet to be fully elucidated, however, as a nuclear factor, the full length of IL-33 is confined in the nucleus of epithelial and endothelial cells [3, 20, 33, 34], consists of up to 270 amino acids, and possesses a homeodomain-like helix-turn-helix DNA-binding domain at the amino-terminal part, the N terminus, which is functional in nuclear localisation, heterochromatin association in the nucleus, and a repressor of transcriptional-based activities upon binding to the chromatin [3, 33, 35]. Furthermore, at the amino terminus part, the full length of IL-33 is also able to interact with the nuclear factor kappa-light-chain-enhancer of activated B cell (NF-*κ*B) transcription factors, p65 subunit [36]. This binding can be enhanced by IL-1*β* in the cytosol and nucleus, which in turn repress the gene activation by reducing the binding to other targets such as tumour necrosis factor alpha (TNF-*α*) and nuclear factor of kappa light polypeptide gene enhancer in B cell inhibitor, alpha (I*κ*B*α*). Besides, IL-33 has been described in deterring regulatory gene transcription by binding with the heterochromatin-associated transcriptional repressor histone lysine methyltransferase SUV39HI [33] and/or acidic pocket of nucleosome of H2A-H2B which induces the nucleosome-nucleosome interaction [35, 37].

IL-33 mediates its proinflammatory properties through the formation of IL-33/ST2L/IL1-RAcP complex [1, 27–29, 38] while inactivated through the conjugation of IL-33, sST2 [5], and SIGIRR [8, 9, 39] or even cleaved by caspases during apoptosis [40–42]. Although it is noted that IL-33 acts as either pro- or anti-inflammatory cytokine, it is also suggested that IL-33 acts as a complex cytokine with features that also appears to be opposing both pro- and anti-inflammatory cytokine as well [43, 44]. IL-33 itself is thought to be as a pro-inflammatory inducer involved in the activation of various cells in the innate immune system, however, recent studies have suggested that IL-33 signalling associated with regulatory T cell (Treg) and regulatory B cell responses [43, 45]. Additionally, another evidence points out the involvement of IL-33 expressed by the brain without an inflammatory response [46]. Mainly, IL-33 induces Th2 innate immune responses as compared with other IL-1 family members [1], though also potentially induces production of Th1 cytokine, IFN-*γ* [30, 47]. Despite this, IL-33 solely acts as a chemoattractant for Th2 cells as opposed to Th1 cells [48]. IL-33 also has been described as an important amplifier for innate rather than adaptive immunity solely [49], which certainly points out as crucial for both innate and adaptive immunity [18, 22, 44, 50, 51]. And in a certain point, IL-33 is being described as “alarmin” due to the cytokine involvement during necrosis and therefore is also associated with damage-associated membrane protein (DAMP) [26].

**Figure 1:**
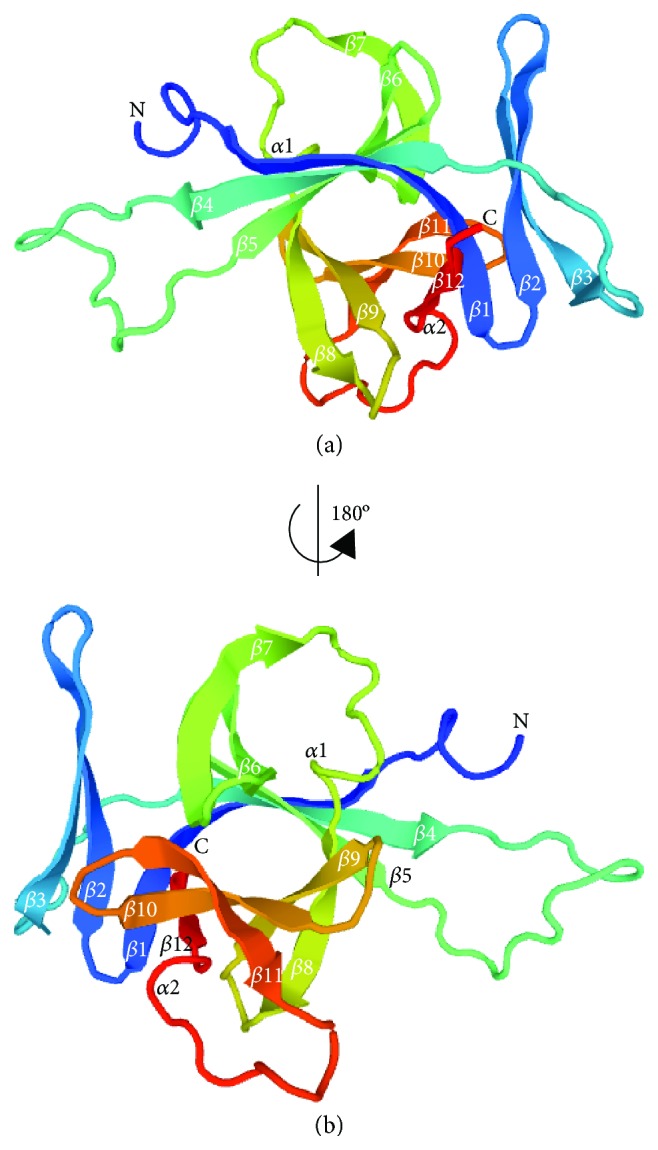
3D molecular structure of IL-33. IL-33 represented in a ribbon diagram with labelled secondary structures. Note the arrangement of the *β*-strands from *β*1 to *β*12 that are arranged from the N- until C-terminus tail (a). (b) The view of (a) correlated by a 180° rotation of the *y*-axis. IL-33 also acts as a ligand which binds to ST2, a high-affinity receptor family member [53]. A ternary complex formation is formed through the IL-33/ST2 complex formation with IL-1RAcP. Furthermore, the designated binding area is somewhat to be both polar and nonpolar regions, which facilitate specific binding between a ligand and a receptor.

IL-33 is classified as one of the members of the IL-1 superfamily due to the sheer similarities of structures based on the *β*-trefoil structure with other members of IL-1 cytokines based on small-angle X-ray-scattering (SAXS) and X-ray crystallography analysis (Figure 1) [32, 52]. IL-33 possesses 12 stranded *β*-trefoil fold, where these 12 *β*-strands form the *β*-trefoil fold and conserved in both structures, while conformational variation residue in the loops containing the strain, chiefly *β*2-*β*3, *β*3-*β*4, *β*4-*β*5, *β*10-*β*11, and *β*11-*β*12 is involved in the receptor interaction. These 12 *β*-strands from the *β*-trefoil folds are arranged in 3 pseudorepeats of 4 *β*-strands units, where the first and last *β*-strands are anti-parallel staves in a 6-stranded *β*-barrel. Meanwhile, each repeat form of the second and third *β*-strands forms a *β*-hairpin sitting atop of the *β*-barrel. As a comparison, IL-33 is identical to IL-18 based on the homologue of the amino acid sequence rather than with other IL-1 family members [1]. Additionally, IL-33 is confined in the nucleus of stromal cells such as IL-1*α* as compared with other IL-1 family members as well [53].

**Figure 2:**
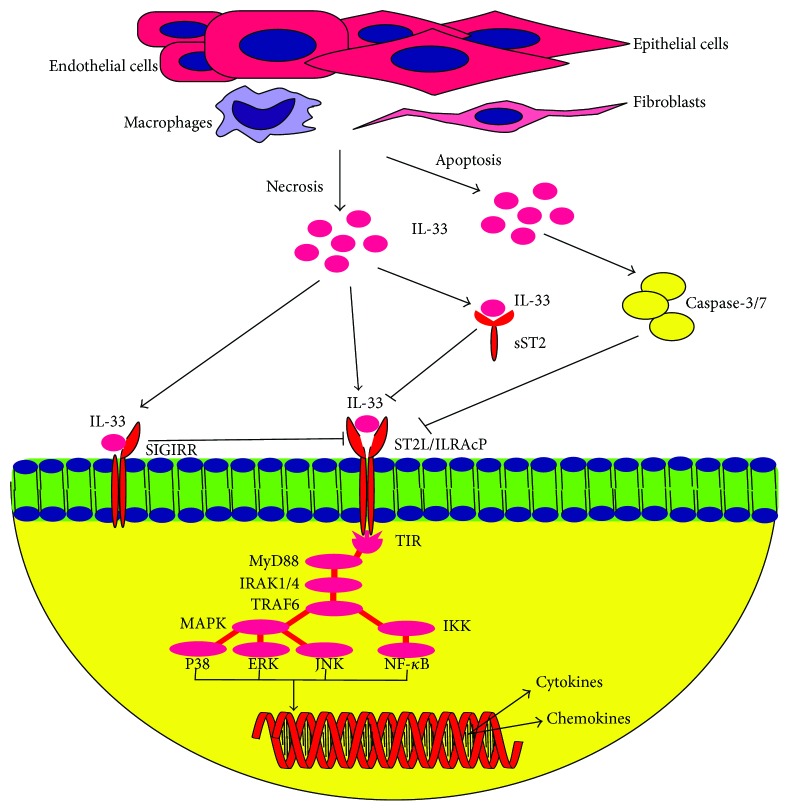
Releases and signalling of IL-33. Full length of IL-33 is predominantly expressed by stromal cells, endothelial and epithelial cells, macrophages, and fibroblasts. In necrosis, IL-33 is released and binds to ST2L/IL1-RAcP heterodimer receptor, which induces signalling via TIR domain of ST2L/IL1-RAcP and recruits MyD88 and followed by IRAK1/4 and TRAF6. TRAF6 further induces either MAPK which in turns activating P38, ERK and JNK, and/or IKK pathway which activate NF-*κ*B. Both pathways lead to the recruitment of transcription factors and proinflammatory cytokines, which consequently drives the regulatory transcription factors of Th2 cytokines and chemokines. Alternatively, binding of IL-33 to sST2 neutralises the proinflammatory effect of IL-33 as well as with SIGIRR which negatively regulates ST2L signalling pathway. In apoptosis, IL-33 is cleaved and deactivated by caspase-3/7.

The full-length IL-33 is mainly expressed by stromal, epithelial, and endothelial cells (Figure 2) [1, 20]. Unlike IL-18 and IL-1*β*, IL-33 does not require proteolytic processing by caspases-1, as a protease, in order to induce signalling via ST2L. Moreover, cleavage activity of caspase-3/7 towards IL-33 deactivates IL-33 and its bioactivities [40, 41]. Unlike Th1 cells, ST2 is expressed by Th2 cells when exposed to IL-33, followed by the secretion of IL-5 and IL-13 by Th2 cells [1, 10, 54, 55]. IL-33-treated mast cells express IL-4, IL-5, and IL-16 [21, 56] while IL-33-treated keratinocytes express IL-6 and TNF-*α* [57].

## 3. Releases and Signalling of IL-33

In the event of necrosis, the release of the full-length IL-33 binds to the heterodimer receptor complex of ST2L/IL-1RAcP, which consists of ST2L (T1, Fit-1, and DER4), a member of the Toll-like receptor (TLR)/IL1R superfamily [1, 27–29]. IL-33 does not form a heterodimer complex with ST2 directly, but rather IL-33 signalling is initiated through the recruitment of an accessory and a coreceptor protein, IL-1R accessory protein (IL1-RAcP), which is necessary to induce ligand-based signalling activation via the dimerisation of both ST2 and IL1-RAcP [29]. There are variants of ST2 receptors, namely, ST2L, a membrane-anchored receptor that responds to IL-33 by heterodimerising with IL-1RAcP [4, 58]; sST2, a decoy and soluble isoform receptor identical to ST2L [29, 59]; ST2V, an isoform receptor similar to sST2 with exception of the third extracellular immunoglobulin domain and can be found in human [6, 60]; and ST2LV, similar with ST2L without transmembrane domain and currently described in chicken, *Gallus gallus*, solely [7]. Signalling is initiated by IL-1RAcP domain via Toll-interleukin-1 receptor (TIR) which leads to the recruitment of MyD88 and subsequently IL-1R-associated kinase 1 or 4 (IRAK1/4) and TRAF6 [1, 61, 62]. TRAF6 further induces signalling of the mitogen-associated protein kinases (MAPK) and IKK pathways, which act as inducers and activate numerous inflammatory mediators and transcription factors such as P38, c-Jun N-terminal kinases (JNK), extracellular signal-regulated kinases (ERK), and NF-*κ*B mediators. These mediators and transcription factors finally drive the production of numerous Th2 cytokines and chemokines [1, 54, 63], which can be derived from several mechanisms. The first mechanism is through the regulation of the gene expression and modulation of chromatin via conjugation of the intracellular IL-33 with the acidic pocket of nucleosome of H2A-H2B [35, 37]. This is achieved through the nuclear IL-33, which is acting as a regulatory transcriptional factor by downregulating the expression of certain receptors and cytokines such as soluble IL-1R4 and IL-6 through the conjugation with transcriptional repressor histone methyltransferase SUV39H [33]. Meanwhile, the second mechanism involves the downregulation of NF-*κ*B and proinflammatory signalling via conjugation of the intracellular IL-33 with NF-*κ*B transcription factors [36].

In the meantime, the binding of IL-33 to sST2 further forms the IL-33/sST2 complex, which sST2 neutralises, and blocks the IL-33 inflammatory activities, thus repressing the NF-*κ*B and Th2 proinflammatory productions [5, 64]. The soluble form, sST2, has been demonstrated to be elevated in several cardiac diseases and therefore considered as a biological marker for the prognosis of the events of cardiac diseases [38, 65, 66]. Inactivation of IL-33 also occurs through the inverse regulation of ST2L signalling by SIGIRR. Meanwhile, in the event of apoptosis, the releases of IL-33 is cleaved and further inactivated by caspase-3/7 via proteolysis [40–42].

## 4. Roles of IL-33/ST2 Pathway in Neurological Disorders

The involvement of IL-33/ST2 pathway has been incriminated in numerous diseases and conditions, such as in asthma and myocardial infarction. However, there are few studies that correlated with the participation of the IL-33/ST2 pathway in the brain, specifically, neurological disorders involving the central nervous system (CNS). ST2L/IL-1RAcP and IL-33 are highly expressed in brain tissues, where IL-33 is constitutively expressed by endothelial cells, as well as by astrocytes, neurones, microglia, and oligodendrocytes while ST2L/IL-1RAcP by microglia, astrocytes, and neurones [1, 17, 67]. Meanwhile, sST2 is expressed by microglia and astrocytes [67]. IL-33 mRNA level was also revealed to be highly expressed in the spinal cord and brain of the mouse [1]. Exposure of microglia with IL-33 produces numerous proinflammatory cytokines such as TNF-*α*, IL-1*β*, and IL-10 and chemokines including CCL2, CCL3, CCL5, and CXCL10 and oxidative stress molecules, namely, nitric oxide (NO) and inducible nitric oxide synthase (iNOS) [67]. Notable and distinct involvement of IL-33/ST2 in neurological disorders is greatly reflected in Alzheimer's disease, multiple sclerosis, and experimental cerebral malaria as summarized in Table 1.

## 5. Alzheimer's Disease

Alzheimer's disease (AD) is a type of neurological disorder characterised by the progressive of cognitive impairment, memory deficits, and personality changes, and this disease causes rapid neurone loss and deteriorations; hence, the aetiology is still unknown and definite treatment is still unavailable [68–71]. Pathophysiologically, it is a form of dementia due to the excessive deposition of extracellular *β*-amyloid (*β*A) plaque and intracellular tau-based neurofibrillary tangles (NFTs) in the brain [72]. Expression of IL-33 mRNA and protein was found to be significantly increased by pathogen-associated molecular patterns (PAMPs) in CNS glia and astrocytes, as well as IL-33 was stimulated and amplified by mast cells which directly induces numerous immune effectors in CNS glia such as arginase I, CCL17, CCL11, and TNF-*α* [19]. Transcriptomic analysis revealed the decrease in IL-33 expression in the brain of Alzheimer's disease cases and this expression is restricted to the endothelium and vascular smooth muscle cells of cell arteries of both AD and non-AD brains [73]. Additionally, overexpression of IL-33 was demonstrated in decreasing the A*β*_40_ peptide secretion, which is one of the main components for the cerebral amyloid angiopathy. Administration of IL-33 induces the proliferation of microglia and fosters the proinflammatory cytokine production such as TNF-*α* and IL-1*β* and anti-inflammatory cytokine IL-10 and the production of chemokines CCL2, CCL3, CCL5, and CXCL10 and phagocytosis of nitric oxide by microglia [67]. High level of ST2 and IL-33 expressions was found to be localised in *β*A plaques, in NFTs, and in the glial cells along with the increase of ST2 and IL-33-positive cells which suggest the involvement of IL-33 as an inflammatory inducer in the case of AD [72]. Intriguingly, the administration and modulation of IL-33 were found to alleviate the neuropathology of AD in mice by reducing the soluble *β*A levels and plaque accumulation through the recruitment of *β*A phagocytic activity and the proinflammatory responses involving of IL-1*β*, IL-6, and NLR family pyrin domain containing 3 (NLRP3) through the polarisation of macrophages/microglia [16]. These further suggest that IL-33 plays a crucial role in the pathogenesis of Alzheimer's disease as a mediator of inflammatory molecules.

## 6. Multiple Sclerosis

Multiple sclerosis (MS) is an autoimmune disease interfering with central nervous system (CNS) and is considered as chronic inflammatory-demyelinating disease due to the involvement of both adaptive and innate immunity responses [74–76]. It is demonstrated that the lesions in MS are comprised of numerous types of leukocytes which are incriminated to be the key factor contributing to the lesion formation [77, 78]. Nevertheless, specific biochemical components involved in leading to the event of MS are yet to be fully understood [79]. In a study utilising experimental autoimmune encephalitis (EAE) as an animal model for MS, IL-33 and ST2 were found to be highly expressed in the spinal cord tissue, while ST2 expression was found to be highly elevated in the spinal cord tissue of EAE-induced mice [80]. EAE has also exacerbated in ST2^−/−^ mice as compared with WT mice, while IL-33-treated WT mice developed impaired EAE. Additionally, it was demonstrated the increases of CD4^+^Foxp3^+^ Treg cells were induced by IL-33 in the lymph nodes and CNS which further suggests the impairment of EAE perhaps due to the Treg cell induction through M2 macrophage induction by IL-33. Furthermore, adoptive transfer experiment of IL-33-treated macrophages impaired the severity of EAE *in vivo*. Meanwhile, the IL-33 level was found to be upregulated in the peripheral and central nervous system of MS patients, and IL-33 plasma level was found to be increased in MS patients as compared with controls; however, IL-33 blood level was found to be significantly decreased in MS patients treated with IFN-*β*-1a [81]. Additionally, IL-33 expression was highly increased in white matter and plaque areas of MS brains. The expression of intracellular IL-33 and IL-33-regulated genes such as OAS1, GATA3, and PMAIP1 was increased in relapsing remitting MS patients (RRMS) [79]. *In vitro* cultivation with TLR agonist lipopolysaccharide (LPS) further demonstrated the increase induction of IL-33 and histone deacetylase (HDAC), an enzyme that is involved in inflammatory regulation, in RRMS patients. Additionally, gene expression from the peripheral blood mononucleated cell (PBMC) culture treated with IL-33 was overlapped with RRMS patients, suggesting that the gene expression observed in RRMS was perhaps regulated by IL-33-mediated immune pathways. An EAE model was also performed and able to demonstrate the significant reduction of intracellular IL-33 as compared with the high increase of extracellular IL-33 in the naïve mice during the preonset, onset, and peak stage of EAE in the spinal cord [25]. The damages of neurones and activation of astrocytes are associated with the reduction of intracellular IL-33 upon *in vitro* inflammatory stimulation during EAE. Additionally, EAE development was exacerbated through the blockage of CNS-derived IL-33 which further suggests the involvement of CNS-derived IL-33 in impairing EAE development. Meanwhile, in both cortex of MS patients and healthy controls, IL-33 was found to be expressed by neurones, astrocytes, microglia, and oligodendrocytes while ST2 was expressed by oligodendrocytes and damaged axons [17]. Intriguingly, the expression of ST2 and IL-33 levels was enhanced in the brain lesions of both acute and chronic MS patients as compared with healthy controls. This further demonstrated the possible inhibition of CNS myelination by IL-33 from established rat CNS-myelinating cocultures, though the involvement of IL-33 in the neurodegenerative process still remains unclear. These evidences suggest that IL-33/ST2 pathway could be involved in the pathogenesis of MS or protective effect against MS.

## 7. Experimental Cerebral Malaria

Malaria is a devastating parasitic infection caused by protozoan *Plasmodium* parasites, transmitted by female *Anopheles* mosquitoes and is endemic in tropical and subtropical areas [82]. According to the World Health Organization (WHO) report, in 2015, approximately 214 million cases and up to 500,000 cases of mortality accounted worldwide with 88% of the cases were comprised of African regions alone [83]. There are many complications manifested due to malaria including cerebral malaria (CM), which is most commonly associated with *Plasmodium falciparum* (*P. falciparum*) malaria infection that often leads to mortality [84]. Pathophysiology of CM is commonly associated with the sequestration of parasitised red blood cells (PRBCs) and inflammatory exudates in the microvessels, along with the exacerbated inflammation in the brain [84–87]. Experimental cerebral malaria (ECM) utilising murine *Plasmodium berghei* (*P. berghei*) ANKA strain (PbA) is widely utilised as a model for the human *P. falciparum* malaria [86–88]. In an ECM study, induction of IL-33 has caused the expansion of type 2 innate lymphoid cells (ILC2), through the type 2 cytokines, such as IL-13, IL-4, and IL-5, and has led to the polarisation of the M2 macrophages and Foxp3 Treg cells [24]. Additionally, Treg-deleted mice treated with IL-33 were unable to resist ECM, while IL-33-treated mice demonstrated significant parasitaemia reduction at the early phase of infection as compared with the untreated mice; however, they succumb to death at the later stage due to the hyperparasitaemia. Meanwhile, no changes of parasitaemia and brain parasite load were found between ST2-deficient and WT mice [15]. The ST2-deficient mice were able to survive more than 20 days without ECM features as compared with the WT mice which succumbed to death at day 10 with ECM, features, such as haemorrhages, brain vascular leakage, and distinct microvascular pathology obstruction. Additionally, the expression of CXCR3, ICAM-1, and LT-*α* was reduced which diminished recruitment and intravascular activation of both activated CD4^+^ and cytotoxic CD8^+^ T-cells which subsequently promote ECM development. Furthermore, recent literature revealed that there were no significant changes in parasitaemia and survival rate between the mutant (IL-25^−/−^, IL-33^−/−^, and TSLP receptor, TSLPR^−/−^) and WT mice [89]. The expression of TSLP mRNA was found to be significantly decreased at the late stage of infections compared with mRNA expression of IL-33 and IL-25 in the brain. Thus, these contradictory findings warrant further investigations as the role mediated by IL-33 in ECM is still unclear at the moment to reflect CM.

## 8. Conclusion

IL-33 has been perceived to render either protective or deleterious role as an important Th2 cytokine inducer in numerous diseases; however, particular role(s) in CNS diseases are yet to be fully understood. It appears that IL-33 renders as a critical inducer and regulator of gene transcriptions and cytokines in innate and adaptive immunity either as an intracellular or extracellular cytokine. In necrosis, the IL-33/ST2 pathway has been demonstrated to be a critical signalling pathway inducing related immune mechanisms, while IL-33/sST2 appeared to be an interesting pathway in neutralising the activation of IL-33. Therefore, further studies are required to elucidate these pathways as they might be beneficial targets in CNS diseases either as novel biomarkers, therapeutic targets, or even as intervention purposes.

## Figures and Tables

**Table 1:** Involvement of IL-33/ST2 pathways in variants of CNS diseases.

Disorder	Findings	References
Alzheimer's disease	(i) IL-33 mRNA and protein expression was increased by PAMPs in CNS glia.	[19]
	(ii) IL-33-stimulated and IL-33-amplified mast cells directly induce arginase I, CCL17, CCL11, & TNF-*α* in CNS glia.	[73]
	(iii) IL-33 expression is reduced in the brains.	
	(iv) IL-33 decreases the secretion of A*β*_40_ peptides when overexpressed.	
	(v) Dose-dependent IL-33 induces proliferation of microglia and stimulates the production of IL-1*β*, TNF-*α*, & IL-10.
	(vi) IL-33 enhanced the production of CCL2, CCL3, CCL5, CXCL10, nitric oxide, and phagocytic activity of microglia.	[67]
	(vii) IL-33 and ST2 expression was highly expressed in *β*A plaque, NFTs, and glial cells in the AD brains.	[72]
	(viii) IL-33 and ST2^+^ cells increased in the brain.	
	(ix) IL-33 reduced soluble *β*A levels and plaque accumulation via *β*A phagocytic activity.	[16]
	(x) IL-33 modulation polarised microglia/macrophages and reduced IL-1*β*, IL-6, & NLRP3 in the brain.	

Multiple sclerosis	(i) IL-33 was highly expressed in peripheral blood and CNS and plague areas in MS brains.	[80, 81]
	(ii) IL-33 plasma level was elevated compared with controls.	[81]
	(iii) IL-33 plasma level was reduced in MS patients treated with IFN-*β*-1a.	
	(iv) Intracellular IL-33 and OAS1, GATA3, & PMAIP1 were highly expressed.	[79]
	(v) IL-33 and HDAC were highly induced in RRMS patients.	
	(vi) IL-33 might be regulating gene expression in RRMS.	
	(vii) IL-33 and ST2 expressions were enhanced in the lesions of acute and chronic MS patients compared to healthy controls.	[17]
	(viii) IL-33 might inhibit CNS myelination.	

Experimental cerebral malaria	(i) IL-33 induces ILC2 expansion via IL-4, IL-5, & IL-13 and polarises M2 macrophages and Tregs.	[24]
	(ii) IL-33-treated Treg^−/−^ mice were unable to resist ECM; IL-33-treated mice reduced parasitaemia at the early phase of infection.	
	(iii) ST2^−/−^ mice and WT mice exhibited no changes in parasitaemia and brain parasite load.	[15]
	(iv) ST2^−/−^ mice survived >20 days compared with WT mice with features of ECM.	
	(v) ICAM-1, CXCR3, and LT-*α* expression reduced and diminished both CD4^+^ and CD8^+^ T-cell recruitment and sequestration.	
	(vi) IL-33^−/−^ and WT mice exhibited no prominent changes in parasitaemia and survival rate.	[89]
	(vii) TSLP mRNA expression was significantly decreased as compared to IL-33 and IL-25 at the late stage of infection.	
